# Low Magnetic Field Exposure Alters Prostate Cancer Cell Properties

**DOI:** 10.3390/biology13090734

**Published:** 2024-09-19

**Authors:** Sigrun Lange, Jameel M. Inal, Igor Kraev, Dafydd Alwyn Dart, Pinar Uysal-Onganer

**Affiliations:** 1 Pathobiology and Extracellular Vesicles Research Group, School of Life Sciences, University of Westminster, London W1W 6UW, UK; 2 Cell Communication in Disease Pathology, School of Human Sciences, London Metropolitan University, 166-220 Holloway Road, London N7 8DB, UK; j.inal@londonmet.ac.uk; 3 Biosciences Research Group, School of Life and Medical Sciences, University of Hertfordshire, Hatfield AL10 9EU, UK; 4 Electron Microscopy Suite, Faculty of Science, Technology, Engineering and Mathematics, Open University, Milton Keynes MK7 6AA, UK; igor.kraev@open.ac.uk; 5 UCL Cancer Institute, University College London, Paul O’Gorman Building, 72 Huntley Street, London WC1E 6DD, UK; a.dart@ucl.ac.uk; 6 Cancer Mechanisms and Biomarkers Research Group, School of Life Sciences, College of Liberal Arts and Sciences, University of Westminster, 115 New Cavendish Street, London W1W 6UW, UK

**Keywords:** prostate cancer, low magnetic/hypomagnetic field, magnetic shielding, extracellular vesicles, proteome, KEGG, miRNA, MMP, cell invasion

## Abstract

**Simple Summary:**

The effects of magnetic fields on health and disease have been subject to considerable research interest for the past few decades but still remain relatively poorly understood. Therefore, the identification of molecular and cellular pathways affected is of considerable importance. This study investigated the effects of very low magnetic field (LMF) exposure in a cellular model of prostate cancer (PCa), the second most common cancer diagnosed in men. Extracellular vesicles (EVs) are lipid structures released from and taken up by cells and play crucial roles in cell communication and in processes such as cancer spread via their protein and nucleic acid cargoes. Short-term (4 h) LMF exposure significantly altered the release profiles and protein content of EVs from PCa cells to a more pro-cancerous profile. We then investigated changes in several key micro-RNAs, which are regulators of cancer behaviour and indicators of cancer aggressiveness and metastasis. LMF exposure caused significant upregulation of three key oncogenic miRNAs (miR-155, miR-21, and miR-210) and significant downregulation of two key tumour-suppressive miRNAs (miR-126 and miR-200c) in the PCa cells. These changes were also associated with a significant increase in the cancer cells’ invasion capability, which is a key indicator of cancer aggressiveness. We further verified the metastatic ability of the cancer cells caused by the LMF exposure by assessing two metastasis-related proteins, matrix metalloproteinases MMP2 and MMP9, which both were significantly increased. We compared these findings with normal prostate cells, which showed fewer changes in response to LMF exposure. Our findings suggest that LMF exposure may promote a more aggressive cancer phenotype by modulating key molecular and cellular pathways, highlighting the potential therapeutic implications of magnetic field modulation in cancer treatment.

**Abstract:**

Prostate cancer is the second most common neoplasia and fifth-leading cause of cancer death in men worldwide. Electromagnetic and magnetic fields have been classified as possible human carcinogens, but current understanding of molecular and cellular pathways involved is very limited. Effects due to extremely low magnetic/hypomagnetic fields (LMF) are furthermore poorly understood. Extracellular vesicles (EVs) are crucial mediators of cellular communication with multifaceted roles in cancer progression, including via transport and uptake of various protein and microRNA (miRNA) EV-cargoes. miRNAs regulate gene expression and are implicated in cancer-related processes such as proliferation, metastasis, and chemoresistance. This study investigated the effects of LMF exposure (20 nT) by magnetic shielding on the prostate cancer cell line PC3 compared to the prostate epithelial cell line PNT2 under short-term (4 h) conditions. We examined EV profiles following a 4 h LMF exposure alongside associated functional enrichment KEGG and GO pathways for the EV proteomes. The 4 h LMF exposure significantly reduced cellular EV release and modified PC3 EV cargoes to a more inflammatory and metastatic profile, with 16 Disease Pathways and 95 Human Phenotypes associated specifically with the LMF-treated PC3 EV proteomes. These included cancerous, metabolic, blood, skin, cardiac and skeletal Disease Pathways, as well as pain and developmental disorders. In the normal PNT2 cells, less EV protein cargo was observed following LMF exposure compared with cells not exposed to LMF, and fewer associated functional enrichment pathways were identified. This pointed to some differences in various cellular functions, ageing, defence responses, oxidative stress, and disease phenotypes, including respiratory, digestive, immune, and developmental pathways. Furthermore, we analysed alterations in matrix metalloproteinases (MMPs) and miRNAs linked to metastasis, as this is crucial in cancer aggressiveness. The 4 h LMF exposure caused a significant increase in MMP2 and MMP9, as well as in onco-miRs miR-155, miR-210, miR-21, but a significant reduction in tumour-suppressor miRs (miR-200c and miR-126) in the metastatic PC3 cells, compared with normal PNT2 cells. In addition, 4 h LMF exposure significantly induced cellular invasion of PC3 cells. Overall, our findings suggest that changes in magnetic field exposures modulate EV-mediated and miR-regulatory processes in PCa metastasis, providing a basis for exploring novel therapeutic strategies.

## 1. Introduction

Prostate cancer (PCa) is the second most prevalent cancer globally, contributing significantly to cancer-related mortality rates [1,2]. Various risk factors, including age, dietary habits, obesity, tobacco and alcohol use, disruptions in circadian rhythms, racial background, and sexual behaviour have been linked to an increased risk of developing PCa. Additionally, environmental and occupational exposures have been suggested to explain the differing epidemiological impacts of the disease across populations [3,4,5].

Previously, a potential association between childhood leukaemia and electromagnetic fields (EMFs) emitted by power lines has been highlighted, leading to their classification as possible human carcinogens [6]. Although the associated risk is considered low, this link was recognised several years ago and is now regarded as a preventable risk factor. However, establishing a definitive causal relationship and elucidating the underlying biological mechanisms has proven challenging, primarily due to limited support from animal and laboratory studies regarding the carcinogenic effects of magnetic, including low (LMF) magnetic fields. Consensus remains elusive on the mechanisms by which magnetic fields interact with biological systems and biomolecules beyond thermal interactions. Moreover, magnetic fields can potentially influence quantum systems within biological molecules, affecting the spin states of electrons and interactions with other molecules. These effects may have implications for various biological processes and enzymatic reactions; however, precise mechanisms by which LMFs interact with cellular processes remain unclear. The emerging field of quantum biology, though in its early stages, is rapidly expanding and gaining recognition. Quantum phenomena have been implicated in a variety of biological processes, including photosynthesis, navigation, enzyme catalysis, olfaction, and DNA mutation [7].

microRNAs (miRs/miRNAs) are single-stranded RNA molecules that can moderate gene expression at the post-transcriptional level and are estimated to control more than 60% of protein-encoding genes [8,9,10]. Furthermore, it has been proposed that ineffective cancer therapy is frequently due to a lack of current understanding of accurate molecular mechanisms that are involved in tumorigenesis. Recently, miRNA profiling and disease-specific miRNA signatures have been widely used for the detection of various cancers, such as pancreatic, prostate, and breast cancers, as they are secreted into body fluids, primarily packaged into EVs [11,12,13,14]. Work in our lab has highlighted the fact that several miRNAs are involved in biological processes such as proliferation, invasion, migration, metastasis, angiogenesis, and chemoresistance [14,15,16,17,18].

Extracellular vesicles (EVs) are 30–1000 nm lipid bilayer-enclosed structures released from cells and taken up by neighbouring cells. EVs play important roles in cellular communication via the transport of EV cargoes, including proteins, genetic material, and non-coding RNAs, some of which are microRNAs. EVs are key mediators in intra- and inter-tumour communication, can influence the tumour microenvironment, participate in the preparation of the pre-metastatic niche, and contribute to cancer aggressiveness [19,20]. The release profiles of EVs from cancer cells are, therefore, of considerable importance, both with respect to EV numbers released as well as EV signatures relating to changes in EV sub-populations and EV cargoes, including proteins and miRs [16,21,22]. Importantly, previous research has shown that exposure to extremely low-frequency strength magnetic fields modulates the numbers of EVs released from various cancer cells *in vitro*, also sensitising some cancer cells to chemotherapeutic treatment [23,24]. 

The current scientific literature lacks a full understanding of mechanisms explaining the interactions between electromagnetic and/or magnetic fields and biological material. Magnetic fields may influence the behaviour of biological molecules at the quantum level via their electron spin states with significant implications for processes where electron transfer is vital, such as mitochondrial ATP production or in enzymatic processes where transient electron spin states are generated. Additionally, magnetic fields may interact with other biomolecules, including DNA and RNA, although the specific physicochemical mechanisms remain unclear [25]. Further research into cellular and molecular mechanisms is, therefore, essential.

Matrix metalloproteinases (MMPs) comprise a group of twenty-four zinc-dependent extracellular endopeptidases, extensively expressed across various tissues and engaged in numerous biological functions. Their primary function is the degradation of all components of the extracellular matrix. Additionally, MMPs play crucial roles in inflammatory processes by modulating the synthesis and release of cytokines and chemokines. Furthermore, MMPs are associated with cellular growth, proliferation, and tissue remodelling [26]. Among these, MMP2 and MMP9 are the most significant cancer-associated zinc-dependent endopeptidases involved in the invasion and metastasis of various carcinomas and elevated expression levels of activated MMP2 or MMP9 have been correlated with metastasis in patients with PCa [27,28].

In this study, we used a known cancer model to examine the impact of LMF/hypomagnetic exposure (20 nT) by magnetic shielding on cell–cell communication and molecular mechanisms involved, focussing on EVs, miRs, and MMPs. We compared the PC3 prostate cancer cell line with the normal prostate epithelial cell line PNT2. We assessed the effects of 4 h magnetic shielding on EV signatures, identifying changes in EV release profiles, total EV protein cargoes, and associated KEGG and GO pathways. Based on those findings, we further assessed changes in key MMPs and selected miRs associated with metastasis. 

## 2. Materials and Methods

### 2.1. Cell Culture

The prostate epithelial cell line PNT2 and the PCa cell line PC3 (ATCC, Manassas, VA, USA) were maintained in Roswell Park Memorial Institute (RPMI) 1640 (Gibco-Life Technologies, Carlsbad, CA, USA) with 10% (*v*/*v*) heat-inactivated foetal bovine serum (FBS; Pan Biotech, Aiedenbach, Germany) and penicillin–streptomycin (10,000 units penicillin/mL and 10 mg streptomycin/mL) (Pan Biotech, Germany) at 37 °C in a humidified 5% CO_2_ incubator (Heracell 150i, Thermo Fisher Scientific, Hemel Hempstead, UK).

### 2.2. Magnetic Shielding—Extremely Low Magnetic/Hypomagnetic Treatment

To investigate the effects of shielding biological samples from Earth’s magnetic field, modelling low magnetic field (LMF) exposure, we utilised an instrument from Magnetic Shields Ltd. (MSL, Staplehurst, Kent, UK), crafted from a metallic alloy known as mu-metal. This material attracts and deflects the geomagnetic field, creating an internal environment of an extremely low magnetic/hypomagnetic (20 nT) field. Cells were plated onto appropriate cell culture plates or flasks for subsequent assays and allowed to adhere overnight. After adherence, the cells were divided into two groups. The experimental LMF group was exposed to the magnetic shield by placing the cells inside the mu-metal instrument at room temperature for 4 h. The control group was kept on the bench outside the incubator at room temperature for the same duration without exposure to the magnetic shield. Following the 4 h exposure period, the cells were processed according to the specific requirements of the assays described below.

### 2.3. RNA Extraction and qRT-PCR

RNA was extracted from cells using Trizol (Sigma, Haverhill, UK), and RNA concentration and purity were measured using the NanoDrop spectrophotometer (Thermo Fisher Scientific, Hemel Hempstead, UK) at 260 nm and 280 nm absorbance. Reverse transcription of RNA to cDNA was carried out using a miRCURY LNA RT Kit (Qiagen, Manchester, UK) according to the manufacturer’s instructions. The miRCURY LNA miRNA SYBR Green (Qiagen, Manchester, UK) was used in conjunction with MystiCq microRNA qPCR primers for miRs 21 (hsa-miR-21-5p MIRAP00047), 155 (hsa-miR-155-5p MIRAP00202), 210 (hsa-miR-210 MIRAP00262), 126 (hsa-miR-126-5p MIRAP00142), and 200c (hsa-miR-200c-5p MIRAP00252), all from Sigma, Haverhill, UK. The resulting cDNA was used to assess the expression of miR-21, miR-155, miR-210, miR126, and miR-200c while RNU6 (F 5′-GCTTCGGCAGCACATATACTAAAAT-3; R 5′-CGCTTCACGAATTTGCGTGTCAT-3′) was used as a reference RNA for normalisation of miRNA expression levels, as described before [11,12]. Each experiment was repeated three times, and the relative expression levels of listed miRNAs were normalised with RNU6 expressions using the comparative cycle threshold method [29]. cDNAs for the analysis of MMP2 (F 5′-GAGAAGACATTCCTCAGAGACG-3′; R 5′-TGGGGAGGTTTACCCTATATGG-3′) and MMP9 (F 5′-GGACCCGAAGCGGACATTG-3′; R 5′-CGTCGTCGAAATGGGCATCT-3′) primers were used as described before and expressions were generated using qScript cDNA Supermix (Quantabio, London, UK) with incubations at 42 °C for 30 min and 85 °C for 5 min [30]. The gene expressions were analysed by using PrecisionPlus qPCR Master Mix (Primer Design, Eastleigh, UK) for RT-qPCR synthesis with the following thermocycling conditions for 40 cycles: 95 °C for 2 min, 95 °C for 10 s, and 60 °C for 60 s. Relative gene expression levels of MMPs were calculated with RNA polymerase II (RPII) (F 5′-GCACCACGTCCAATGACAT-3′ R; 5′-GTGCGGCTGCTTCCATAA-3′) as described before [11,18].

### 2.4. EV Isolation and Characterisation 

EV isolation was carried out according to established and previously published protocols [21,22], also adhering to the recommendations of the International Society of Extracellular Vesicle Research (ISEV; MISEV2023) [31]. Cells were cultured to a 70% confluence in T25 flasks, and the adherent cells were washed with sterile-filtered EV-free Dulbecco’s Phosphate-Buffered Saline (DPBS) before applying 5 mL of fresh cell culture medium per flask, omitting foetal bovine serum (FBS) for the duration of the 4 h experiment, to avoid contamination with FBS derived EVs. Negligible detection of EVs in the cell-free medium was confirmed by NTA. Following the 4 h LMF incubation (the control treatment was handled the same way but kept outside the LMF chamber), EVs were isolated from the cell culture supernatants (from the 3 flasks per experiment, containing 5 mL medium each) from each T25 flask as follows: First the supernatants were centrifuged at 4000× *g* for 30 min at 4 °C to remove cell debris, whereafter the supernatant was carefully collected by pipetting and centrifuged for 1 h at 100,000× *g* at 4 °C for the enrichment of total EVs, generating an EV pellet. The supernatants were carefully aspirated and discarded, and the isolated EV pellets were thereafter resuspended and washed in ice-cold sterile-filtered EV-free DPBS and centrifuged again at 100,000× *g* for 1 h at 4 °C. The final EV-enriched pellets were then resuspended in 100 μL sterile-filtered EV-free DPBS for further analysis. nNanoparticle tracking analysis (NTA) was carried out using the NS300 Nanosight (Malvern Panalytical Ltd., Malvern, UK), equipped with a sCMOS camera and a 405 nm diode laser, to enumerate the EVs and assess EV size profiles. Samples were diluted 1:100 in sterile-filtered EV-free DPBS, and the number of particles in the field of view was maintained in the range of 30–50 with a minimum concentration of samples at 5 × 10^7^ particles/mL. The camera settings for recording were set at 13 and for post-processing of videos at setting 5, according to the manufacturer’s instructions (Malvern Panalytical Ltd.). Five 60 s videos were recorded per sample, and the replicate histograms were averaged using the NTA 3.4 software. Each experiment was repeated in three biological replicates. EVs were further characterised by the two surface markers CD63 (ab216130, Abcam, Cambridge, UK) and Flotillin-1 (ab41927, Abcam) using western blotting and imaged by transmission electron microscopy (TEM), according to previously published protocols [21,22]. Briefly, for TEM, EVs pellets were resuspended in 0.1 M sodium cacodylate buffer (pH 7.4), and a 3–5 µL drop of EVs suspension was applied onto a glow-discharged carbon film-supported TEM grid. After allowing the suspension to air dry for approximately 10 min, the grid was placed sample-side down onto a drop of 2.5% glutaraldehyde fixative solution (Agar Scientific Ltd., Stansted, UK) in 0.1 M sodium cacodylate buffer (pH 7.4) for 1 min at room temperature. The grid was then washed by placing it onto three separate drops of distilled water, removing excess water between each step using filter paper. Next, the grid was placed onto a drop of 2% aqueous uranyl acetate (Agar Scientific Ltd., Stansted, UK) for 1 min for staining. Excess stain was removed using filter paper, and the grid was air dried. Imaging of the EVs was performed using a JEOL JEM 1400 microscope (JEOL, Tokyo, Japan) operated at 80 kV, with magnifications ranging from 10,000× to 30,000×, with digital images captured using a 16-megapixel GATAN RIO 16 camera (AMETEK (GB) Limited, Leicester, UK). 

### 2.5. Liquid Chromatography with Tandem Mass Spectrometry (LC-MS/MS) and STRING Protein–Protein Interaction Network Analysis

EV protein cargoes from isolated EV preparations of LMF exposed and control/untreated PC3 and PNT2 cells, respectively, were analysed for protein hits by LC-MS/MS. EVs were isolated from 3 × 5 mL culture medium as described above, from three T25 flasks per experimental group. Proteins were verified by SDS-PAGE and silver staining before running the EV protein isolates 0.5 cm into a 12% TGX gel and cutting each sample out as one band. The gel bands were then subjected to in-gel digestion followed by LC-MS/MS by Cambridge Proteomics (Cambridge, UK). In brief, automated LC-MS/MS analysis was carried out using a Dionex Ultimate 3000 RSLC nanoUPLC (Thermo Fisher Scientific Inc., Waltham, MA, USA) system in conjunction with a QExactive Orbitrap mass spectrometer (Thermo Fisher Scientific Inc., Waltham, MA, USA). Peptide separation was carried out using reverse-phase chromatography and a Thermo Scientific reverse-phase nano Easy-spray column (Thermo Fisher Scientific Inc). The LC eluent was sprayed into the mass spectrometer using an Easy-Spray source (Thermo Fisher Scientific Inc.). The *m/z* values of all eluting ions were measured in an Orbitrap mass analyser; data-dependent scans (selecting top 20) were employed for automatic isolation and generation of fragment ions using the HCD collision cell, measured using the Orbitrap analyser. Both singly charged ions as well as ions with unassigned charge states were excluded from selection for MS/MS. A dynamic exclusion window of 20 sec was also applied. Data were processed post-run using Proteome Discoverer (version 2.1., Thermo Scientific), converted to mgf files, and submitted to Mascot (Mascot search algorithm; Matrix Science, London, UK). Search for hits was carried out against the UniProt *Homo_sapiens*_20221011 database (226,953 sequences; 74,609,178 residues) with peptide and fragment mass tolerances respectively set at 20 ppm and 0.1 Da. The threshold value for significance was set at *p* < 0.05, and the peptide cut-off score was set at 35. To generate protein–protein interaction networks and associated functional enrichment pathway analysis, protein hits were fed into the STRING database (https://string-db.org/; accessed 19 April 2024) and analysed based on the *Homo sapiens* database. Settings were at medium confidence. Protein–protein interaction networks were generated in STRING for each experimental group and compared between the control and LMF-treated EV proteomes. STRING functional enrichment pathway analysis was used to identify shared and distinct Gene ontology (GO), Reactome, and STRING cluster pathways, as well as Disease–gene associations and Human Phenotype. Functional enrichment tables were downloaded from STRING as Excel files and the protein–protein interaction network images were downloaded as PNG files.

### 2.6. Assays for Cellular Invasion and Proliferation

Cell invasion assay was performed as follows: 5 × 10^5^ cells were plated on Matrigel-coated transwell filters (Corning™ BioCoat™ Matrigel™ Invasion Chamber with Corning™ Matrigel Matrix; BD Biosciences, Wokingham, Berkshire, UK) in a chemotactic gradient of 1:10% FBS. After 4 h incubation either inside of the magnetic shield instrument (LMF) or outside as the control, the total number of invaded cells was determined by MTT assay (Abcam, Cambridge, UK) and further confirmed by crystal violet assay (Abcam, UK). In parallel, the same number of cells was plated and incubated for 4 h to determine the effect of LMF exposure on cell proliferation by MTT (3-(4,5-dimethylthiazol-2-yl)-2,5-diphenyl tetrazolium bromide) assay. Absorbance was measured using CLARIOstar plate reader (BMG Labtech, Aylesbury, UK) at 540–590 nm and normalised according to the control (*n* = 3).

### 2.7. Data Analysis 

All data were checked for normal distribution and analysed as means ± standard deviation (SD). Statistical significance was determined using a Student’s *t*-test or ANOVA with a Newman–Keuls post hoc analysis, as appropriate. Results were considered significant for *p* < 0.05. One-way ANOVA Bonferroni’s multiple comparisons test was performed using GraphPad Prism version 7.00 for Windows (GraphPad Software, La Jolla, CA, USA). 

## 3. Results

In summary, this study determined the effects of 4 h magnetic shield (low magnetic/hypomagnetic) exposure on the prostate cancer PC3 cell line, compared to the normal (immortalised and nontumourigenic) prostate epithelial cell line PNT2. EV profiling was carried out for changes in EV numbers released and on EV protein cargoes. Based on these outcomes, further assessments were carried out for metastasis-associated MMPs and miRNAs, as well as changes in PC3 cell invasion and proliferation capacities. 

### 3.1. EV Profiles from PC3 and PNT2 Cells Were Modified in Response to 4 h LMF Exposure

**Figure 1:**
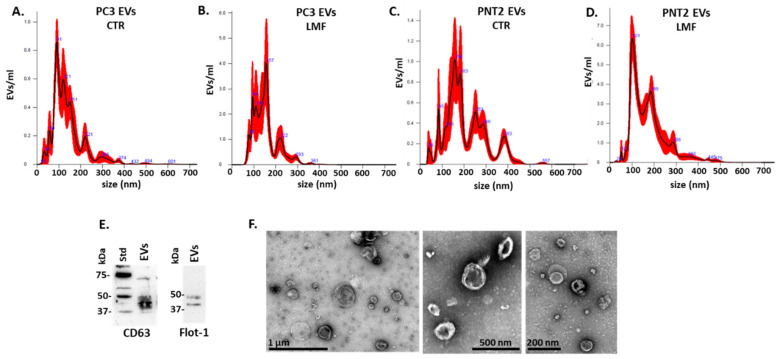
EVs isolated from PC3 and PNT2 cells. (**A**–**D**) NTA profiles of PC3 and PNT2 EVs, comparing control and 4 h magnetic field shielding (LMF). (**E**) Western blotting confirms two EV-specific surface markers, CD63 and Flotillin-1 (see Supplementary Figure S1 for full western blot). (**F**) Representative transmission electron microscopy (TEM) images of isolated EV are shown with scale bars indicated in µm or nm.

EVs obtained from PC3 and PNT2 cells were isolated by differential centrifugation and characterised using nanoparticle tracking analysis (NTA, representative figures are shown in Figure 1A–D), two EV-specific surface markers (CD63 and flotillin-1) by western blotting (Figure 1E) and transmission electron microscopy (TEM) (Figure 1F). 

**Figure 2:**
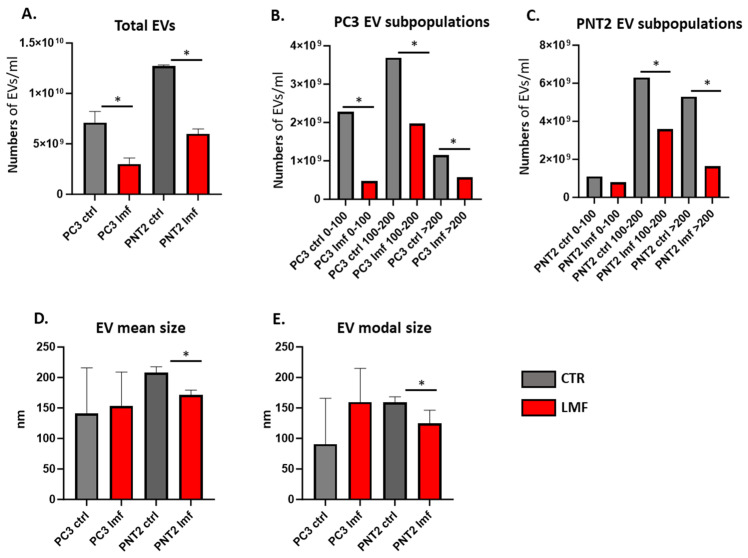
EV profiling from PC3 and PNT2 cells, comparing control and 4 h LMF exposure. (**A**) Total EV numbers released from the cells; (**B**) EV subpopulations from PC3 cells, control and LMF exposed; (**C**) EV subpopulations from PNT2 cells, control and LMF exposed; (**D**) mean size of EVs released from PC3 and PNT2 cells, showing controls and LMF exposed; (**E**) modal sizes of EVs from PC3 and PNT2 cells, showing control and LMF exposed; * *p* < 0.05.

EVs were enumerated by NTA, assessing total EV numbers released from PC3 and PNT2 cells under control and LMF conditions (Figure 2A). Differences in EV subpopulations released were measured, considering small EVs ≤ 100 nm, medium EVs 101–200 nm, and large EVs > 200 nm (Figure 2B,C). Also, the mean size (Figure 2D) and modal size (Figure 2E) of EVs were assessed. Following 4 h LMF exposure, both PC3 and PNT2 cells showed a significant reduction in total EV numbers released (Figure 2A), and all EV sub-populations were significantly reduced following LMF exposure in PC3 cells (Figure 2B). In PNT2 cells, the numbers of medium-sized EVs (101–200 nm) and large EVs (>200 nm) were significantly reduced following LMF exposure (Figure 2C). EV mean size was reduced in PNT2 but not changed in PC3 cells following 4 h LMF exposure (Figure 2D). EV modal size showed a trend in increase (but not significantly) in PC3 LMF-treated cells, while EV modal size was reduced in PNT2 cells following LMF exposure (Figure 2D).

### 3.2. Proteomic EV Cargoes Showed a Shift to More Pro-Cancerous Signature in PC3 Cells Following 4 h LMF Exposure

**Figure 3:**
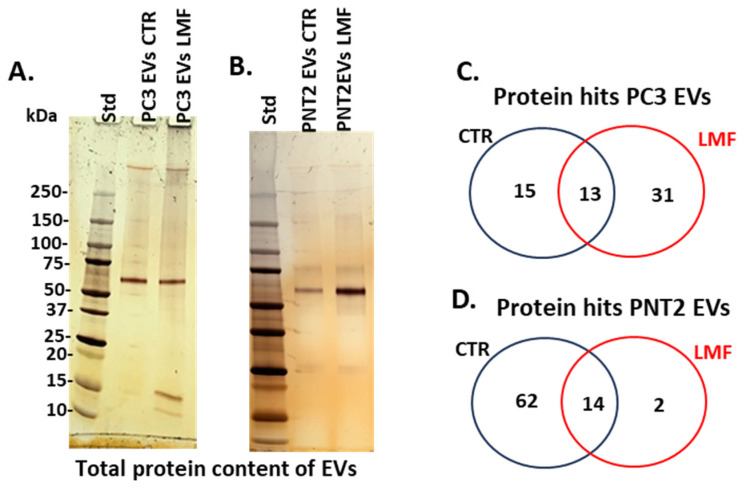
Proteomic analysis of EV cargoes from 4 h LMF-treated (and control untreated) PC3 and PNT2 cells. (**A**) EV protein content, as assessed by SDS-PAGE and silver staining, from PC3-derived EVs, showing control untreated (CTR) and LMF-treated cell-derived EVs. (**B**) Protein content from PNT2-derived EVs, showing control untreated (CTR) and LMF-treated cell-derived EVs. (**C**,**D**) Venn diagrams showing shared and specific proteins identified via LC-MS/MS in the EVs of PC3 (**C**) and PNT2 (**D**) cells, respectively, comparing control conditions to the 4 h LMF treatment.

EVs were isolated from the PC3 and PNT2 cell cultures according to methods described in Section 2.4. The protein content of EVs was assessed by SDS-PAGE with silver-staining for PC3-derived EVs from the control and LMF-treated cells (Figure 3A) and from PNT2-derived EVs from the control and LMF-treated cells, respectively (Figure 3B). Proteomic content was then analysed by LC-MS/MS, and numbers of shared and unique protein hits per experimental group are presented in the Venn diagrams for PC3 EVs (Figure 3C) and PNT2 EVs (Figure 3D), respectively (for full information on LC-MS/MS analysis see Supplementary Tables S1–S4). 

The protein hits identified for EV cargoes from PC3 and PNT2 cells, comparing cell-derived EVs from control untreated to LMF exposed cells, respectively, are listed and summarised in Table 1. Proteins that were identified in the EV proteomes of the PC3 LMF treated cells only included Keratin II, Keratin 6A, Actin cytoplasmic 2, Immunoglobulin heavy chain variable region, Annexin 1, Haemoglobin subunit beta, Phosphopyruvate hydratase, Villin 2/Ezrin, Histone H2A.Z, Histone H2B, Serine protease 1, Fructose-bisphosphate aldolase, Interferon-induced transmembrane protein, Dermcidin, CD44 antigen, Triosephosphate isomerase, HSP90AA1 protein, Ventricular zone-expressed PH domain-containing protein, and Tyrosine 3-monooxygenase/tryptophan 5-monooxygenase activation protein eta.

### 3.3. Protein–Protein Interaction Network Analysis for EV Protein Cargoes, Comparing LMF Treated to Control Untreated PC3 Cells

**Figure 4:**
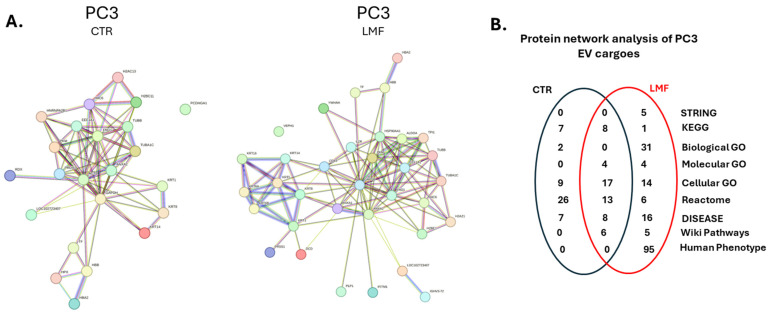
Protein–protein interaction network analysis for PC3-derived EV protein cargoes. (**A**) Protein interaction networks are shown for EV protein cargoes from PC3 control and PC3 LMF-treated cells, respectively. (**B**) The Venn diagram summarises numbers of shared and group-specific functional enrichment pathways associated with the EV proteomes from control and LMF-treated PC3 cells. For a full list of pathways, see Table 2.

Protein–protein interaction networks for EV protein cargoes were created in STRING (https://string-db.org/; accessed 19 April 2024) for the EVs isolated from PC3 cells, the control, and 4 h LMF treatment, respectively. A considerable change was observed in the protein interaction networks and associated functional enrichment pathway analysis for the PC3-derived EVs following 4 h LMF exposure (Figure 4A). Furthermore, Gene ontology (GO) analysis showed increased pathways associated with EV proteomes of LMF-treated cells (Figure 4B), including 31 Biological GO, 4 Molecular GO, and 14 Cellular GO pathways only identified following LMF treatment. In addition, an increase in disease-associated pathways was observed, with 16 DISEASE and 95 Human Phenotype (Monarch) pathways identified for protein cargoes of the EVs from LMF-treated PC3 cells. Details on the protein network annotations for the PC3-derived EVs are listed in Table 2. 

### 3.4. Protein–Protein Interaction Network Analysis for EV Protein Cargoes, Comparing LMF Treated to Control Untreated PNT2 Cells

**Figure 5:**
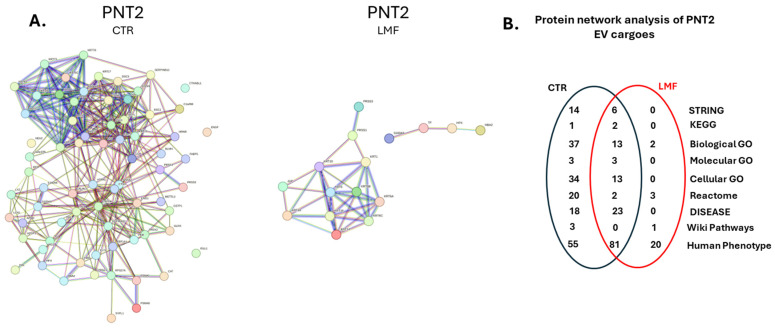
Protein–protein interaction network analysis for PNT2-derived EV protein cargoes. (**A**) Protein–protein interaction networks are shown for EV protein cargoes from PNT2 control untreated and PNT2 LMF-treated cells, respectively. (**B**) The Venn diagram summarises numbers of shared and group-specific functional enrichment analysis pathways associated with the EV proteomes from control untreated and LMF-treated PNT2 cells. For a full list of pathways, see Table 3.

Protein–protein interaction networks for EV protein cargoes were created in STRING for the EVs isolated from PNT2 cells, from control and 4 h LMF treatment, respectively. Considerably fewer proteins were identified in the EV proteome of the LMF-treated PNT2 cells, as reflected in the differences in the protein-interaction networks (Figure 5A). Furthermore, functional enrichment analysis pathways were accordingly associated with the control EV proteome, compared to the EV proteome of the LMF-treated cells. This included 37 biological and 34 Cellular GO pathways, as well as 18 DISEASE and 55 Human Phenotype pathways for the control EV proteome. For the EV proteome of LMF-treated PNT2 cells, 30 Human Phenotypes were specific. Furthermore, several pathways were identified in both groups, as summarised in the Venn diagram in Figure 5B. For details on pathways, see Table 3.

**Figure 6:**
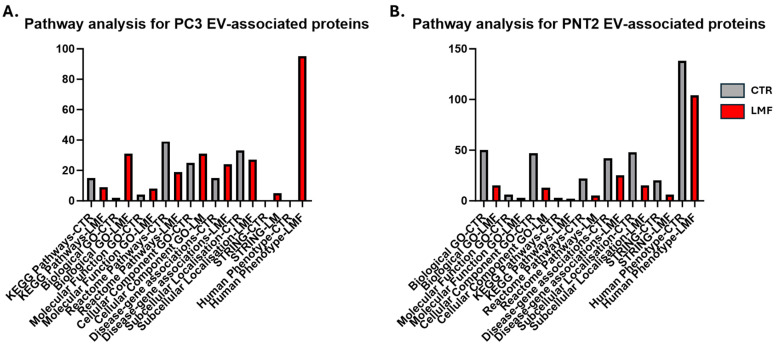
Summary for the functional protein enrichment analysis of protein cargoes identified in EVs derived from (**A**) PC3 and (**B**) PNT2 cells, following 4 h LMF exposure (in red), compared with controls (in grey).

A summary of the PC3 and PNT2 EV proteome-associated protein-interaction networks and functional enrichment analysis are presented in Figure 6. This shows Biological GO, Molecular Function GO, Cellular Component GO, KEGG and Reactome pathways, Disease–gene associations, Subcellular localisations, STRING and Human Phenotype pathways for PC3-EV-associated proteins (Figure 6A) and PNT2-EV-associated proteins (Figure 6B), respectively, indicating LMF-treated groups in red. 

### 3.5. Expression Levels of Oncogenic and Tumour Suppressor miRNAs Were Differently Modulated in Response to LMF Exposure, Only in PC3 Cells

**Figure 7:**
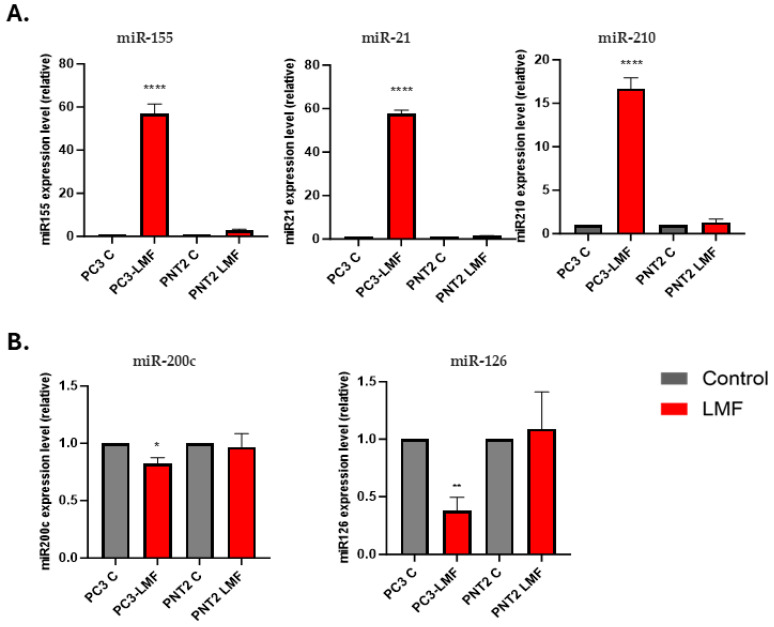
Comparative reverse transcription–quantitative polymerase chain reaction (RT-qPCR) analysis of expression levels of miR-155, miR-210, miR-21, miR-200c, and miR-126. (**A**) Relative expressions of oncogenic miRs: miR-155, miR-21, miR-210 in PNT2 and PC3 cells following 4 h LMF exposure. (**B**) Relative expression levels of tumour suppressor miRs: miR-200c and miR-126 in PNT2 and PC3 cells following 4 h LMF exposure. The column graphs represent the average of three replicates of RNA isolated from each sample. Data were normalised according to RNU6 expression by fold analysis (*n* = 3, *p* < 0.05 for all). Exact *p*-values are indicated (* *p* ≤ 0.05; ** *p* ≤ 0.01; **** *p* ≤ 0.0001); error bars indicate standard deviation (SD).

Following 4 h LMF exposure, effects on key oncomiRs and tumour suppressor miRs were investigated. Based on our previous and other studies, miR-21, miR-210, miR-155, miR-200c, and miR-126 are closely associated with the development of PCa at different stages [10,12,32,33,34,35]. When assessing oncomiRs expression levels (miR-155, miR-210, miR-21) and tumour-suppressor miRs (miR-200c and miR-126) comparing control with LMF-exposed cells, significant expression changes were observed in response to LMF exposure only in the metastatic PC3 cell line but not in the non-tumorigenic control PNT2 cell line (Figure 7A,B). Following 4 h LMF exposure, miR-155 showed a 57-fold increase (*p* < 0.0001) in PC3 cells, compared to only a 2-fold (ns) increase in PNT2 cells; miR-21 was 57-fold increased (*p* < 0.0001) in PC3 cells, while no significant change was observed in PNT2 cells, and miR-210 showed a 17-fold increase in PC3 cells (*p* < 0.0001), while no significant change was observed in PNT2 cells (Figure 7A). The 4 h LMF exposure resulted in a 1.25-fold decrease in tumour-suppressor miR-200c (*p* < 0.05) and a 2.5-fold decrease in miR-126 (*p* < 0.01) in PC3 cells, but no significant changes were observed for the PNT2 cells (Figure 7B).

### 3.6. LMF Exposure Enhanced the Expression Levels of Matrix Metalloproteinases (MMP2 and MMP9) in PC3 Cells

**Figure 8:**
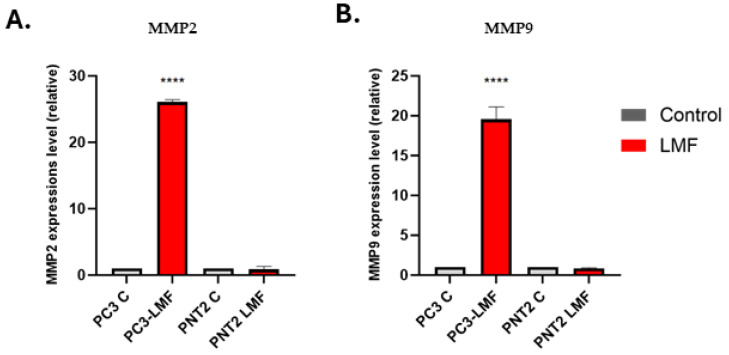
Comparative reverse transcription–quantitative polymerase chain reaction (RT-qPCR) analysis of expression levels of MMP2 and MMP9. (**A**) Relative expressions of MMP2 in PNT2 and PC3 cells following the LMF exposures. (**B**) Relative expression level of MMP9 in PNT2 and PC3 cells following the LMF exposures. The column graphs represent the average of three replicates of RNA isolated from each sample. Data normalised according to RNU6 expression by fold analysis. (*n* = 3, *p* < 0.05 for all). Exact *p*-values are indicated (**** *p* ≤ 0.0001); error bars indicate standard deviation (SD).

Following the EV proteome data analysis, which indicated an increase in metastasis-related proteins, we analysed the expression levels of MMP2 and MMP9 after exposing both cell lines to 4 h LMF treatment. We found that LMF exposure induced MMP2 and MMP9 mRNA levels significantly (*p* < 0.0001) in the PC3 cells by 25-fold and 20-fold, respectively; however, no significant changes were detected in the PNT2 cells (Figure 8A,B).

**Figure 9:**
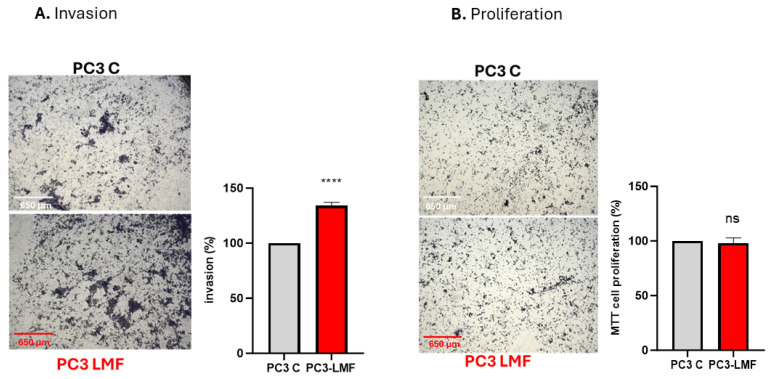
LMF exposure (4 h) induced cellular invasion of PC3 cells but did not affect cell proliferation. (**A**) PC3 cells were plated on Matrigel-coated transwell filters, and the extent of invasion was determined following a 4 h LMF exposure and compared to the control assay. The results are plotted as invasion (%), which is the percentage of invaded cells compared to the total number of cells seeded *(n* = 3; **** *p* ≤ 0.0001). (**B**) The total cell number/proliferation did not change during the experiment (*n* = 3; ns); error bars indicate SD. Scale bars indicate 650 μm for all images that are representative of triplicates.

To confirm the effects of LMF exposure on PCa metastasis, a Matrigel invasion assay was carried out. The assay was conducted during a 4 h LMF exposure time window inside the magnetic field shield instrument at room temperature and compared to a control assay, without LMF exposure, also carried out at room temperature. Despite using a shorter incubation period of 4 h rather than a typical overnight incubation, the PC3 cells demonstrated significantly increased invasion (34% increase, *p* < 0.0001) when kept in the LMF chamber for 4 h, compared to cells kept outside the effects of LMF (Figure 9A). In parallel, a proliferation assay was performed, with no changes detected in cellular proliferation (Figure 9B).

## 4. Discussion

PCa remains the second leading malignancy and the fifth cancer-related cause of death among men worldwide, with approximately 1.4 million new cases and 400,000 deaths [36]. Given its high incidence and mortality, this malignant disease represents an important public health problem [37]. Magnetic field effects and the influences of exposure to altered magnetic conditions are gaining increased attention in biological research [38], including in cancer [39]. To date, while a range of studies has been carried out, understanding of mechanisms is still limited, including exact roles in the regulation of cellular processes. Furthermore, short-term and long-term effects on mechanisms involved in cancer are relatively poorly understood. Therefore, studies identifying molecular and cellular pathways influenced by changes in magnetic field exposure are of great importance enhancing the current understanding of their contribution to disease processes and identifying possible therapeutic benefits. 

The current study focussed on assessing the effects of a short (4 h) low magnetic/hypomagnetic field exposure (20 nT, using a magnetic shield instrument) on prostate cancer cell properties *in vitro*. Effects of such low magnetic field exposure (LMF) were assessed for changes in extracellular vesicle (EV) signatures, focussing on proteomic cargoes, with results indicating pro-metastatic changes. Therefore, further investigations focussed on cancer-associated MMPs as well as key oncogenic and tumour-suppressor miRNAs. Matrigel cell invasion assay confirmed that PC3 cell invasion increased in response to the 4 h LMF exposure. PC3 cell proliferation capacity was also assessed in response to the 4 h LMF treatment. 

EVs were significantly reduced following a 4 h LMF exposure, and this was observed for small (<100 nm), medium (101–200 nm), and large (>200 nm) EVs in the PC3 cells, indicating that all EV subpopulations were influenced by LMF exposure. This may have considerable effects on cellular communication in prostate cancer, as generally increased EV release is associated with cancers, and EVs contribute significantly to metastatic processes [40,41]. Interestingly, a reduction in EV numbers was also observed for the PNT2 cell line, with a significant reduction in medium and large EVs but not small EVs. The different EV subpopulations have been subject to a wide range of studies, with a focus on small EVs and medium/large EVs in various cancer models in response to different treatments, while studies on the effects of changes in magnetic fields are very limited to date [23,24]. Therefore, the current findings are of considerable importance to gain an understanding of the roles of EV modulation in cellular communication in response to LMF exposure. Interestingly, previous research showed the enhanced release of calcium-stimulated EV release (medium/large EVs) from human monocytic leukaemia cells following 30 min pulsed LMF treatment at 0.3 μT [23]. This indicates possible differences between EV release profiles in response to LMF treatment between cancer cell lines, but also that LMF levels and time windows of LMF exposure may cause different effects of EV release. Importantly, changes in EV cargoes must also be considered, as EVs carry a range of protein, non-coding RNA, genetic, and other cargo. The focus of the current study was, therefore, also on EV protein cargoes, and interestingly, a considerable change in protein hits and increased protein hits were identified in PC3-derived EVs following the 4 h LMF exposure. While 13 protein hits were common between control and LMF-treated EV cargoes, 15 were unique to the controls, but there were 31 hits unique to EVs of the LMF-treated PC3 cells. Proteins that were only identified in the EV proteomes of the PC3 LMF-treated cells included Actin cytoplasmic 2, Cytoskeletal Keratins (Type I 16, Type II 5, Type II 6A and B), Immunoglobulin heavy chain variable region, Annexin 1, Haemoglobin subunit beta, Phosphopyruvate hydratase, Villin 2/Ezrin, Histone H2A.Z, Histone H2B, Serine protease 1, Fructose-bisphosphate aldolase, Interferon-induced transmembrane protein, Dermcidin, CD44 antigen, Triosephosphate isomerase, HSP90AA1 protein, Ventricular zone-expressed PH domain-containing protein, and Tyrosine 3-monooxygenase/tryptophan 5-monooxygenase activation protein eta. Several of these have been associated with cancer pathology, including metastasis, aggressiveness, and chemoresistance, also in PCa, and are briefly discussed below. Actins play multifaceted roles in podosomes and invadopodia formation [42]. Various keratins, including *KRT5* and *KRT6A,* have been associated with PCa assessment and prognosis [43,44]. Ezrin has been identified as an indicator of metastasis via EV export [45] and as a circulating biomarker for PCa metastasis [46]. Histone H2A.Z is linked to the regulation of tumorigenesis, metastasis, and response to chemotherapy [47,48], while post-translational modifications of H2B are implicated in cancer initiation and progression [49]. Annexin 1 is a reported proteomic marker of PCa metastasis [50]. Fructose-bisphosphate aldolase has been identified in EV proteomes linked to PCa chemoresistance [51]. Interferon-induced transmembrane protein 1 belongs to a group of IFITM proteins that have been implicated in cancer aggressiveness and chemoresistance [52]. Dermcidin is associated with cancer survival, including in PCa [53]. CD44 is a cancer stem cell marker and indicative of PCa tumour initiation, drug resistance, metastasis, and recurrence [54]. *HSP90AA1* is involved in PC3 necroptosis via mitochondrial fission pathways and ROS generation [55]. Tyrosine 3-monooxygenase/tryptophan 5-monooxygenase activation protein eta (*YWHAH*) is linked to EV-mediated activation of cancer-associated fibroblasts [56].

In relation to the EV proteomes, there was a marked increase in the numbers of associated functional protein pathways, based on STRING analysis. This included 5 STRING pathways and 1 KEGG pathway, and an increase in GO pathways with 31 Biological, 4 Molecular, and 14 Cellular GO pathways unique to the PC3 LMF-treated EVs. Interestingly, 16 Disease Pathways and furthermore 95 Human Phenotypes were unique to the PC3 LMF-treated EVs. These were linked to stress, cytoskeletal function, nitric oxide, antimicrobial activity, immune function (including the complement system), hypoxia responses, cancer, histone acetylation, and metabolism. Some of these will be discussed in relation to the published literature below. 

Previous studies using magnetic shielding have, for example, studied the roles of hypomagnetic conditions in stimulating the proliferation of neural progenitor and stem cells associated with observed central nervous system dysfunction and developmental abnormalities in animals [57]. This correlates to some of the functional enrichment pathways identified in our current study. For example, the link to cardiac conditions identified here correlates to cardiovascular studies under hypomagnetic conditions in humans [58], changes in erythrocytes in a rat model [59], increase in human blood hemolysis [60], and increased embryo mortality and modified cardiac function, also associated to the circadian rhythm, in zebrafish [61]. Changes in immune response pathways relate to some published studies, including increased blood granulocytes [62] and increased neutrophil respiratory burst [63]. Effects on cytoskeleton organisation have been reported in cellular cancer models, relating to pathways identified here in relation to LMF exposure in PC3 cells [62]. Effects of hypomagnetic fields on DNA methylation have been reported in embryonic stem cells [64], and this pathway was identified here as associated with the control PC3 EV proteome but not following LMF exposure. Various defence pathways and bacterial pathways were identified in the EV proteomes. Some were shared between control and LMF-treated conditions. Effects of hypomagnetic field exposure have been reported, for example, on antibiotic resistance [65,66]. 

Interestingly, in the PNT2-derived EVs, considerably fewer protein hits were identified following LMF exposure compared to controls. This indicates a shift in protein export via EVs in normal cells in response to LMF effects and may be of considerable relevance for understanding influences on normal cellular processes. Notably, there was also a loss of many functional pathways associated with the changes in the EV proteomes of PNT2 cells following LMF exposure. For example, 14 STRING and 1 KEGG pathways were associated with the control cell EVs, but these were not present in the LMF-treated ones, while 6 and 2 other pathways were shared between both groups. A similar pattern was observed for GO pathways, with 37 Biological, 3 Molecular and 34 Cellular GO pathways associated with the controls, and further 13,3 and 13 shared, respectively. In the LMF-exposed PNT2 cells, there were only two unique Biological GO pathways. Furthermore, Reactome pathways showed 20 unique for the control, two shared, and three unique for the LMF-treated group. A considerable difference was also seen for Disease pathways, with 23 shared pathways but no unique ones for the LMF-treated group, while 18 unique ones were associated with the control EV PNT2 proteome. Human Phenotypes similarly indicated 81 shared pathways, 55 unique for the control PNT2 EV proteomes, but fewer, 20, for the LMF PNT2 EV proteomes. Pathways unique to the PNT2 LMF-treated group were related to corticotropin-releasing hormone signalling pathway, hematopoietic system disease, hemidesmosome assembly, cell junction organisation, and the uptake of dietary cobalamin into enterocytes. Human Phenotypes identified for the LMF group related to digestive, skeletal, musculoskeletal, conjunctiva, mucosal abnormalities, squamous cell carcinoma, various skin disorders, and furthermore, respiratory stress, and abnormal temperature regulation. This indicates that LMF exposure does add to some cellular stress responses. The loss of so many functional enrichment pathways may also indicate that many critical pathways may be negatively affected by LMF treatment in normal cells. It may also be postulated that the absence of various biological process pathway associations, including ageing, response to oxidative stress, cell differentiation, and immune response pathways, may have some positive effects on normal cells. Such speculations will require further investigations as effects of changes in magnetic fields will have multifaceted effects, and further time windows and ranges of fields will need to be explored, both on multiple cell types *in vitro*, as well as using *in vivo* models. 

Interestingly, changes to oxidative stress regulation by reduced ROS were previously reported in response to hypomagnetic field exposure in rat hippocampal neurogenesis [67]. In some cancer cells, oxidative stress has been reported to be reduced in hypomagnetic conditions [68]. This may contribute to carcinogenic effects, which are reported in response to low magnetic fields [69]. In this context, different hypomagnetic conditions and time windows must also be considered. Our findings on identified functional networks also relate to studies reporting changes in skeletal muscle functioning, muscle metabolism regarding glucose and glycogen [57,70], and bone functioning/fragility [71]. Various developmental pathways were identified here as modified in EV cargoes. Previous studies in various models have shown links to hypomagnetic effects on developmental processes, including gamete quality, embryogenesis, teratogenic effects, and malformations [72,73,74,75]. Pain was one of the associated pathways identified for the EV proteome, and changes in pain sensitivity have been reported in mollusc models in response to electromagnetic fields [76]. While digestive-associated functional networks were identified in EV proteomes in this study, previous studies have reported no hypomagnetic effects on water and food intake in mice [62], but further studies will most likely be needed, both comparing different models as well as different LMF exposures. 

Exposure of PC3 cells to LMF led to upregulation in miR-155, miR-21, and miR-210 in the current study. miR-155 is associated with inflammatory responses and has been linked to enhanced tumour growth and metastasis [77]. miR-21 promotes oncogenesis by targeting tumour-suppressor genes, thereby facilitating cell survival and proliferation [11,17]. miR-210, often upregulated under hypoxic conditions, aids cancer cells in adapting to low-oxygen environments and is correlated with increased tumour aggressiveness [78]. Conversely, LMF exposure of PC3 cells resulted in the current study in the downregulation in miR-126 and miR-200c. miR-126 inhibits angiogenesis by targeting VEGF signalling pathways, thereby suppressing tumour growth and metastasis [79]. Similarly, miR-200c plays a role in regulating EMT by targeting the transcription factors *ZEB1* and *ZEB2* [13,80]. Interestingly, LMF exposure did not result in significant changes in the expression of these miRNAs in PNT2 cells. The LMF-induced modulation of both oncogenic and tumour-suppressive microRNAs observed here in PC3 cells suggests that LMF exposure may contribute to a more aggressive cancer phenotype by influencing gene expression pathways associated with tumour progression. Further studies will be required to elucidate the underlying mechanisms and explore the potential of these microRNAs as therapeutic targets in PCa.

As some of the PC3 EV cargo protein hits in the LMF group indicated differences in metastatic associated pathways, further assessment was also carried out for selected key MMPs. *MMP2* and *MMP9* were both confirmed to be upregulated in PC3 cells following 4 h LMF exposure, while no significant changes were seen in the PNT2 cells. High expression levels of activated *MMP2* or *MMP9* have been associated with metastasis in patients with PCa [27,28,81,82]. Studies have indicated that serum levels of matrix MMP2 are correlated with the grading and malignancy of PCa [27,83]. It was suggested that MMP2 could be used as a molecular marker for PCa and may serve as a predictive indicator for the disease [84]. Additionally, the expression levels of *MMP2*, influenced by the regulation in associated pathways, have been shown to either promote or inhibit tumour cell invasion in PCa [85]. Elevated MMP9 expression was reported in PCa patients compared to those with benign prostatic hyperplasia [86,87]. Higher MMP9 expression levels are associated with an increased metastatic rate, and its inhibition may reduce the metastatic potential of PCa [88]. MMP9 plays a crucial role in multiple stages of cancer development, including reducing cancer cell apoptotic potential, promoting angiogenesis, and modulating the immune response to cancer cells [89]. However, the precise mechanism by which hormonal therapy influences MMP9 expression levels remains unclear, necessitating further investigation. In this current study, analysis of the EV cargo proteomics data, indicating a more aggressive signature, correlates with the findings that MMP2 and MMP9 mRNA levels were enhanced following the 4 h LMF exposure in PC3 cells. Indeed, the Matrigel invasion assay confirmed that LMF exposure increased cellular invasion capabilities while proliferation did not change within the 4 h exposure time.

Previous studies on hypomagnetic treatment of neuroblastoma cells showed wide-ranging changes in decreased gene expression associated with cell survival and cell death regulation [90]. It is also of interest that in neuronal models, hypomagnetic exposure induced proliferation rate [57] and this was also observed for neuroblastoma cells [91]. This may vary between cell types, as no effects on normal endothelial cells were observed in other studies [92]. Extremely low-frequency electromagnetic fields of 0.3 μT have been reported to reduce cancer cell migration, increase proliferation, and enhance uptake in cytotoxic drugs in PC12, THP-1, and HeLa cancer cell lines following 30 min LMF treatment [24]. However, proliferation was not affected in the PC3 or PNT2 cells in our current study following 4 h LMF (20 nT) exposure. Further time windows and ranges of LMF exposure may need to be explored in future studies to fully understand the effects of short- and longer-term LMF, as well as ranging LMF exposures on different cancer cells and cancer-type properties. 

The effects of magnetic fields on health and disease are of great interest, and investigations into therapeutic benefits as well as disease risk are required to understand the complex phenomena associated with their still relatively poorly understood functions [93]. Furthermore, interest in utilising magnetic-based therapies in cancer includes, for example, the development of magnetic nanoparticles [94,95,96]. Various limitations for studies on the effects of changed magnetic field exposure, including low magnetic and hypomagnetic conditions, on cellular and organismal systems cannot be ignored and will need attention to move the field forwards. This includes different experimental setups, types of devices used, time windows and levels of exposure, and additional effects, including radical pairs and interference quantum effects [38]. These variables may make it hard to compare experiments between studies and also hamper the repeatability of experiments between research groups. Differing sensitivity of cellular mechanisms to magneto-biological effects may also vary between cell types and organ systems [93], and studies have indeed reported different outcomes between cell types, including cancer types, as also mentioned in our discussion above. Furthermore, the translatability from cellular to organismal level may be an additional challenge, although comparisons between some studies, including our current findings with the wider literature, are encouraging in this aspect. Influences of hypomagnetic conditions on living organisms and the increased interest in this research field, also in relation to future space missions, have recently been extensively reviewed by Sarimov et al. [38]. Further research in both *in vitro* and *in vivo* models is, therefore, of high priority and will aid the future standardisation and optimisation of methods. Importantly, in our current study, we report that even a short (4 h) exposure to magnetic shielding significantly modified cellular EV release profiles and induced notable molecular changes in pro-metastatic pathways at the protein and nucleic acid levels. Research into molecular and cellular mechanisms, including our findings here, will contribute to furthering the current understanding of hypomagnetic conditions at both cellular and organismal levels. This may aid in identifying disease-associated, but possibly also health-promoting, mechanisms with clinical relevance. 

## 5. Conclusions

This study identified novel molecular and cellular communication mechanisms affected by short-term low magnetic/hypomagnetic exposure by magnetic field shielding, with significant effects on cancer cells. The findings of this study indicated that short-term 4 h LMF (20 nT) exposure/magnetic shielding induced significant changes in prostate cancer cell properties *in vitro*. More pro-cancerous changes were observed in EV profiles, oncogenic miRNA expressions, and cellular invasion capabilities of PC3 cells without affecting proliferation. EV protein content was modified to a more pro-inflammatory and cancerous signature following 4 h LMF exposure, based on functional enrichment analysis. This correlated with the upregulation in oncogenic miRNAs miR-155, miR-21, and miR-210, alongside the downregulation in tumour-suppressive miRNAs, miR-126 and miR-200c, suggesting that LMF exposure may promote a more aggressive cancer phenotype by modulating gene expression pathways associated with tumour progression. Additionally, a significant increase in metalloproteinase *MMP2* and *MMP9* expression, which are linked to enhanced metastatic potential, further underscored the potential of LMF to influence PC3 cancer cell behaviour. Our findings report new mechanisms of LMF-induced cellular, molecular, and epigenetic changes and highlight the need to explore the potential implications of modulated magnetic field exposure on human health. Our study also emphasises the importance of investigating the differential effects of LMF on normal cells, as evidenced by the distinct EV proteome changes observed in PNT2 cells. Improving current understanding of downstream mechanisms due to altered magnetic fields is important in medical research, including cancer biology, with putative implications for future therapeutic strategies as biological reactions in cancer cells may be adapted to normal MF strength, and MF shielding may influence cellular behaviour.

## Figures and Tables

**Table 1:** Protein hits identified in EVs isolated from PC3 cells, from control untreated (ctrl) and 4 h LMF exposure groups, respectively. A tick (V) indicates that the protein hit was present in the EV proteome. Protein IDs and names are shown, and additionally, gene names are included for some hits as indicated in italics.

Protein ID	Protein Name	PC3 ctrl	PC3 LMF	PNT2 ctrl	PNT2 LMF
H6VRG0 *KRT1*	Keratin, type II cytoskeletal 1		V		
H6VRG2 *KRT1*	Keratin, type II cytoskeletal 1		V	V	V
P02533	Keratin, type I cytoskeletal 14	V	V	V	V
P08779	Keratin, type I cytoskeletal 16		V	V	V
P13647	Keratin, type II cytoskeletal 5		V	V	V
B4E1T1 *KRT5*	cDNA FLJ54081, highly similar to Keratin, type II cytoskeletal 5		V	V	
P04259	Keratin, type II cytoskeletal 6B		V		V
A0A0S2Z428	HCG2039812, KRT6A		V	V	V
A0A804GS07	Actin, cytoplasmic 2		V	V	
Q6GMX6 IGH@	IGH@ protein	V	V		
P0DOX5	Immunoglobulin gamma-1 heavy chain		V	V	
A0A384NYT8 *TUBB*	Tubulin beta chain	V	V		
P05787-2 *KRT8*	Isoform 2 of Keratin, type II cytoskeletal 8	V	V	V	
A0A0K0K1H8 *HEL-S-71p*	Serotransferrin	V	V	V	V
A0A5E4	Uncharacterised protein		V		
A0A024R5Z7	Annexin 2	V	V	V	
A0A4D5RA86 *ANXA1*	Annexin 1		V		
A0A087WVQ9	Elongation factor 1-alpha		V	V	
A0A087WV01	Elongation factor 1-alpha	V			
A0A5C2GAZ2	IGH + IGL c262_heavy_IGHV3-15_IGHD4-17_IGHJ4	V	V		
Q2TSD0 V9HVZ4 *GAPDH*	Glyceraldehyde-3-phosphate dehydrogenase	V	V	V	
A0A0B4J1Y9	Immunoglobulin heavy variable 3–72		V		
B3KPS3 *TUBA1C*	Tubulin alpha chain	V	V		
A0A2R3Z0D6 *HBB*	Haemoglobin subunit beta		V		
Q0VAS5 *HIST1H4H*	Histone H4	V	V		
A0A7S5BYV3	IGH c429_heavy_IGHV1-24_IGHD1-7_IGHJ4		V		
E2DRY6 *ENO1*	Phosphopyruvate hydratase		V		
A0A2U8J951 *IgH*	Ig heavy chain variable region		V		
B2R6J2 *VIL2*	cDNA, FLJ92973, highly similar to Homo sapiens villin 2 (ezrin) (*VIL2*), mRNA		V		
Q0KKI6	Immunoglobulin light chain (Fragment)	V	V		
A0A286YES1 *IGHG3*	Immunoglobulin heavy constant gamma 3		V		
P0C0S5	Histone H2A.Z		V	V	
H0Y8D1	Serine protease 1		V	V	V
A0A0M4FNU3 *ALDOA*	Fructose-bisphosphate aldolase		V		
A0A0K2BMD8 *HBA2*	Mutant haemoglobin alpha 2 globin chain	V	V		V
A0A024R5Z9	Pyruvate kinase	V	V	V	
A0A0S2Z4D4	Proteolipid protein 1 isoform 1		V		
A0A024R210 *IFITM1*	Interferon-induced transmembrane protein 1 (9–27)		V		
A0A2R8Y619	Histone H2B type 2-K1		V		
P81605-2 *DCD*	Isoform 2 of Dermcidin		V	V	
A0A385KNS5 *CD44*	CD44 antigen		V		
Q2QD09 *TPI1*	Triosephosphate isomerase		V		
Q2VPJ6 *HSP90AA1*	HSP90AA1 protein		V		
Q14D04-2 *VEPH1*	Isoform 2 of Ventricular zone-expressed PH domain-containing protein homolog 1		V		
A0A024R1K7	Tyrosine 3-monooxygenase/tryptophan 5-monooxygenase activation protein, eta polypeptide		V		
H6VRF8 *KRT1*	Keratin, type II cytoskeletal 1	V		V	V
A0A2R8Y793	Actin, cytoplasmic 1	V			
P0C0S8	Histone H2A type 1	V			
A0A024R4F1	Phosphopyruvate hydratase	V		V	
P0DOY2	Immunoglobulin lambda constant 2	V			
A0A481SHK9 *HBB*	Haemoglobin subunit beta	V			
A0A024RCJ2	Histone H2B	V			
A0A024RA28 HNRNPA2B1	Heterogeneous nuclear ribonucleoprotein A2/B1	V			
Q9BS19	Epididymis secretory sperm binding protein	V			
A0A0A0MRQ5	Peroxiredoxin-1	V			
A0A286YFJ8	Immunoglobulin heavy constant gamma 4	V			
A0A5C2GPU9	IG c1228_heavy_IGHV3-33_IGHD1-1_IGHJ4	V			
Q9Y5H4-2 *PCDHGA1*	Isoform 2 of Protocadherin gamma-A1	V			
A0A2R8Y5P0	Radixin	V			
P48668	Keratin, type II cytoskeletal 6C, *KRT6C*			V	V
P15924	Desmoplakin			V	
B4DKV4 *KRT6B*	cDNA FLJ60647, highly similar to Keratin, type II cytoskeletal 6B			V	
Q04695	Keratin, type I cytoskeletal 17, *KRT17*			V	
Q86YZ3	Hornerin			V	
Q02413	Desmoglein-1			V	
P12035	Keratin, type II cytoskeletal 3			V	
A0A0C4DGB6	Albumin			V	
Q0IIN1 *KRT77*	Keratin 77			V	
A0A024R952	Plakophilin 1 (Ectodermal dysplasia/skin fragility syndrome)			V	
Q86Y46	Keratin, type II cytoskeletal 73			V	
Q8N1N4	Keratin, type II cytoskeletal 78			V	
A0A494C0J7	TGc domain-containing protein			V	
A0A1U9X8X5 *CDSN*	Corneodesmosin			V	
Q9HB00	Desmocollin 1			V	
Q5D862	Filaggrin-2, *FLG2*			V	
Q5K634	SCCA2/SCCA1 fusion protein isoform 1			V	
P05089-2 *ARG1*	Isoform 2 of Arginase-1, *ARG1*			V	
Q3SYB5 *SERPINB12*	Serpin B12			V	
P47929	Galectin-7, *LGALS7B*			V	
A0A087WYS6	Proteasome (Prosome, macropain) subunit, alpha type, 8			V	
A0A384P5Q0	Catalase			V	
E7DVW5	Fatty acid binding protein 5 (Psoriasis-associated)			V	
P05109	Protein S100-A8			V	
A0JNT2 *KRT83*	KRT83			V	
B4DF70 *PRDX2*	cDNA FLJ60461, highly similar to Peroxiredoxin-2			V	
Q6KB66-3 *KRT80*	Isoform 3 of Keratin, type II cytoskeletal 80, *KRT80*			V	
P42357-2 *HAL*	Isoform 2 of Histidine ammonia-lyase			V	
A0A024RC29 *DSC3*	Desmocollin 3			V	
B0AZM8 *TGM1*	cDNA, FLJ79468, highly similar to Protein-glutamine gamma-glutamyltransferase K			V	
A0A2R8YD45	Tripeptidyl-peptidase 1, *TPP1*			V	
Q9NZT1	Calmodulin-like protein 5			V	
A0A7P0TAI0 *HSPA5*	78 kDa glucose-regulated protein			V	
A0A0B4J259	Lysozyme C			V	
J3KSD8	Bleomycin hydrolase (Fragment)			V	
A0A2R8Y5E5	Glutathione S-transferase P, *GSTP1*			V	
A0A248RGE3 *RPS27A*	Ubiquitin-40S ribosomal protein S27a (Fragment)			V	
A0A024RD80	Heat shock protein 90kDa alpha (Cytosolic), class B member 1, *HSP90AB1*			V	
A0A024R8D7	Lipocalin 1 (Tear prealbumin), isoform CRA_a			V	
A0A087WVQ6	Clathrin heavy chain, *CLTC*			V	
A0A0K2BMD8 *HBA2*	Mutant haemoglobin alpha 2 globin chain			V	
A0A087WUB9	Beta-catenin-like protein 1, *CTNNBL1*			V	
A0A0S2Z3L4 *CTSD*	Cathepsin D isoform 2 (Fragment), *CTSD*			V	
Q5T750	Skin-specific protein 32			V	
A0A0U1RQT9	Synaptophysin-like protein 1 (Fragment), *SYPL1*			V	
Q9Y3R4	Sialidase-2, *NEU2*			V	
B4DGC3 *APOD*	Apolipoprotein D			V	
F5GX11	Proteasome subunit alpha type-1, *PSMA1*			V	
A0A024RAM2	Glutaredoxin (Thioltransferase)				
P0DOX8 *IGL1*	Immunoglobulin lambda-1 light Chain, *IGL1*			V	
B4DTN4	N6-adenosine-methyltransferase catalytic subunit, *METTL3*			V	
A0A140VK00	Testicular tissue protein Li 227			V	
A0A1B4WRL5 *HBB*	Beta globin (Fragment)			V	
B3VL17	Beta globin (Fragment)				V
A0A1B0GVI3 *KRT10*	Keratin, type I cytoskeletal 10 *KRT10*			V	V
P02790	Hemopexin			V	V
B4DE59 A0A024R1X8 *JUP*	Junction plakoglobin			V	V
A1A508 *PRSS3*	PRSS3 protein			V	V
Q86W19 *PRSS1*	Protease serine 1 (Fragment), *PRSS1*				V
A0A024RAY2	Keratin 18				V
B2R4M6 *S100A9*	Protein S100			V	V

**Table 2:** Functional enrichment pathway analysis for EV protein cargoes derived from PC3 cell, control untreated, and LMF-treated groups, respectively. A tick (V) indicates that the pathway was present for the EV proteome.

STRING Cluster Pathways	PC3 ctrl	PC3 LMF
Pachyonychia congenita and Epidermolysis bullosa simplex Dowling–Meara type		V
Formation of the cornified envelope and Serpin, conserved site		V
Phosphoglycerate kinase and Aerobic glycolysis		V
Glycolysis and sugar phosphatase activity		V
Carbon metabolism and Pyruvate metabolism		V
**KEGG Pathways**	**PC3 ctrl**	**PC3 LMF**
African trypanosomiasis	V	V
Glycolysis/Gluconeogenesis	V	V
Malaria	V	
Systemic lupus erythematosus	V	V
Biosynthesis of amino acids	V	V
Viral myocarditis	V	
HIF-1 signalling pathway	V	V
Phagosome	V	
Pathogenic *E.coli* infection	V	V
Carbon metabolism	V	V
Alcoholism	V	
Tight junction	V	
Viral carcinogenesis	V	V
Salmonella infection	V	
Amyotrophic lateral sclerosis	V	
*S. aureus* infection		V
**Biological Process GO**	**PC3 ctrl**	**PC3 LMF**
Hydrogen peroxidase catabolic process	V	
Retina homeostasis	V	
Nitric oxide transport		V
Positive regulation of plasminogen activation		V
Positive regulation of vesicle function		V
Canonical glycolysis		V
Glycolytic process		V
Killing of host by symbiont cells		V
Intermediate filament organisation		V
Keratinisation		V
Keratinocyte differentiation		V
Complement activation		V
Glucose metabolic process		V
Hexose metabolic process		V
Antimicrobial humoral response		V
Humoral immune response		V
Epidermis development		V
Supramolecular fibre organisation		V
Defence response to bacterium		V
Epithelial cell differentiation		V
Monocarboxylic acid metabolic process		V
Cytoskeleton organisation		V
Defence response to other organism		V
Innate immune response		V
Epithelium development		V
Response to other organism		V
Defence response		V
Immune response		V
Immune system process		V
Response to external stimulus		V
Organelle organisation		V
Response to stress		V
Cellular component organisation		V
**Wiki Pathways**		
Aerobic glycolysis	V	V
Glycolysis and gluconeogenesis	V	V
Glycolysis in senescence	V	V
Metabolic reprogramming in colon cancer	V	V
Pathogenic Escherichia coli infection	V	V
Clear cell renal cell carcinoma pathways	V	V
Cori cycle		V
Sudden infant death syndrome (SIDS) susceptibility pathways		V
VEGFA-VEGFR2 signalling		V
HIF1A and PPARG regulation of glycolysis		V
Corticotropin-releasing hormone signalling pathway		V
**Molecular Function GO**	**PC3 ctrl**	**PC3 LMF**
Structural constituent of cytoskeleton	V	V
Cadherin binding	V	V
Structural molecule activity	V	V
Protein binding	V	V
Phospholipidase A2 inhibitor activity		V
Structural constituent of skin epidermis		V
Disordered domain-specific binding		V
Protein dimerization activity		V
**Cellular Component GO**	**PC3 ctrl**	**PC3 LMF**
Haptoglobin–haemoglobin complex	V	V
Haemoglobin complex	V	V
Endocytic vesicle lumen	V	V
CENP-A containing nucleosome	V	
Blood microparticle	V	
Ficolin-1-rich granule lumen	V	V
Nuclear matrix	V	
Nucleosome	V	
Keratin filament	V	V
Cortical cytoskeleton	V	
Secretory granule lumen	V	V
Cell cortex	V	
Endocytic vesicle	V	
Chromosomal region	V	
Extracellular exosome	V	V
Secretory granule	V	V
Polymeric cytoskeletal fibre	V	V
Supramolecular fibre	V	V
Extracellular space	V	V
Vesicle	V	V
Cytoplasmic vesicle	V	V
Cytoskeleton	V	V
Intracellular non-membrane-bounded organelle	V	
Cytosol	V	V
Protein containing complex	V	V
Extrinsic component of external side of plasma membrane		V
Immunoglobulin complex, circulating		V
M band		V
Cornified envelope		V
Myelin sheath		V
Blood microparticle		V
Intermediate filament	V	V
Basal plasma membrane		V
Basolateral plasma membrane		V
Extrinsic component of membrane		V
Collagen-containing extracellular matrix		V
Apical plasma membrane		V
Side of membrane		V
Cell surface		V
Intracellular non-membrane bound organelle		V
**Disease–Gene Associations**	**PC3 ctrl**	**PC3 LMF**
Amyloidosis	V	V
Cutaneous T-cell lymphoma	V	
non-Hodgkin lymphoma	V	V
Primary cutaneous amyloidosis	V	
Familial visceral amyloidosis	V	V
Skin carcinoma	V	
Borst–Jadassohn intraepidermal carcinoma	V	V
Hematopoietic system disease	V	V
Carcinoma	V	V
Seborrheic keratosis	V	V
Mycosis fungoides	V	V
Alpha thalassemia	V	
Blood protein disease	V	
Organ system cancer	V	
Hepatocellular carcinoma	V	
Inherited metabolic disorder		V
Alpha thalassemia		V
Keratosis		V
Palmoplantar keratosis		V
Pachyonychia congenita		V
Nonepidermolytic palmoplantar keratoderma		V
Congenital haemolytic anaemia		V
Primary cutaneous amyloidosis		V
Autosomal dominant disease		V
Epidermolysis bullosa simplex Dowling–Meara type		V
Epidermolysis bullosa simplex with mottled pigmentation		V
Focal nonepidermolytic palmoplantar keratoderma		V
Basal cell carcinoma		V
Skin disease		V
Steatocystoma multiplex		V
Blood protein disease		V
**Reactome Pathways**	**PC3 ctrl**	**PC3 LMF**
Erythrocytes take up oxygen and release carbon dioxide	V	V
Scavenging of heme from plasma	V	V
Erythrocytes take up carbon dioxide and release oxygen	V	V
Chaperone mediated Autophagy	V	V
Prefoldin-mediated transfer of substrate to CCT/Tric	V	
RHO GTPases activate IQGAPs	V	
Recyclin pathway of L1	V	
RNA polymerase I promoter opening	V	
Gluconeogenesis	V	V
Packaging of telomere ends	V	
DNA methylation	V	
Activated PKN1 stimulates transcription of androgen receptor	V	
Gene and protein expression by JAK-STAT signalling	V	
SIRT1 negatively regulates rRNA expression	V	
Cleavage of damaged purine	V	
Recognition and association of DNA glycosylase with site containing an affected purine	V	
B-WICH complex positively regulates rRNA expression	V	
HDACs deacetylate histones	V	
Assembly of the ORC complex at the origin of replication	V	
Diseases of programmed cell death	V	
Glycolysis	V	V
HCMV early events	V	
HATs acetylate histones	V	
HCMV late events	V	
Formation of the cornified envelope	V	V
Autophagy	V	V
Factors involved in megakaryocyte development and platelets	V	
RHO GTPase effectors	V	
Neutrophil degranulation	V	V
M phase	V	V
Cellular responses to stress	V	V
Vesicle-mediated transport	V	
Infectious disease	V	
Hemostasis	V	
Developmental biology	V	V
Innate immune system	V	V
Disease	V	
Post-translational protein modification	V	
Immune system	V	
Type I hemidesmosome assembly		V
HSF1 activation		V
Binding and uptake in ligands by scavenger receptors		V
RHO-GTPases activate PNKs		V
Metabolism of carbohydrates		V
Immune system		V
**Human Phenotype**	**PC3 ctrl**	**PC3 LMF**
Palmoplantar blistering		V
Blistering by anatomical location		V
Onychogryphosis		V
Lower limb pain		V
Nail dystrophy		V
Hyperhidrosis		V
Palmoplantar keratoderma		V
Hoarse voice		V
Nail dysplasia		V
Steatocystoma multiplex		V
Eruptive vellus hair cyst		V
Abnormal fingernail morphology		V
Linear arrays of macular hyperkeratosis in flexural areas		V
Hypohidrosis or hyperhidrosis		V
Neoplasm of the skin		V
Onychogryphosis of toenails		V
Abnormality of the digestive system		V
Angular cheilitis		V
Hyperplastic callus formation		V
Onychogryphosis of fingernail		V
Paronychia		V
Abnormality of skin morphology		V
Pain		V
Abnormality of temperature regulation		V
Palmoplantar hyperhidrosis		V
Constitutional symptom		V
Epidermoid cyst		V
Abdominal symptom		V
Neoplasm by anatomical site		V
Pain		V
Ear pain		V
Thickened skin		V
Sign or symptom		V
Fingernail dysplasia		V
Oral leukoplakia		V
Follicular hyperkeratosis		V
White lesion of the oral mucosa		V
Jaundice		V
Mottled pigmentation of the trunk and proximal extremities		V
Discrete 2 to 5 mm hyper- and hypopigmented macules		V
Alopecia		V
Abnormality of digestive system physiology		V
Focal friction-related palmoplantar hyperkeratosis		V
Generalised reticulate brown pigmentation		V
Localised skin lesion		V
Natal tooth		V
Pain in head and neck region		V
Cholestasis		V
Punctate palmoplantar hyperkeratosis		V
Skin fragility with non-scarring blistering		V
Smooth tongue		V
Foot pain		V
Genital blistering		V
Hyperkeratotic papule		V
Fever		V
Acute episodes of neuropathic symptoms		V
Reticulated skin pigmentation		V
Depigmentation/hyperpigmentation of skin		V
Aplasia cutis congenita on trunk or limbs		V
Hypomelanotic macule		V
Generalised abnormality of skin		V
Abnormality of the skeletal system		V
Chronic haemolytic anaemia		V
Erythematous papule		V
Dermatological manifestations of systemic disorders		V
Upper limb pain		V
Cholelithiasis		V
Spotty hyperpigmentation		V
Spotty hypopigmentation		V
Diffuse palmoplantar hyperkeratosis		V
Erosion of oral mucosa		V
Abnormal circulating protein concentration		V
Lower limb amyotrophy		V
Abnormal hair quantity		V
Nonspherocytic haemolytic anaemia		V
Lamina lucida cleavage		V
Abnormality of blood and blood-forming tissues		V
Feeding difficulties		V
Anaemia of inadequate production		V
Abnormal oral mucosa morphology		V
Anaemia		V
Abnormal skeletal morphology		V
Oral mucosal blisters		V
Decreased body weight		V
Female reproductive system disease		V
Cutaneous photosensitivity		V
Abnormal hair morphology		V
Abnormality of the respiratory system		V
Congestive heart failure		V
Absent toenail		V
Abnormality of the immune system		V
Cholecystitis		V
Abnormality of the musculoskeletal system		V
Ovarian endometrioid carcinoma		V
Normocytic anaemia		V

**Table 3:** Functional enrichment pathway analysis for EV protein cargoes derived from PNT2 cells, control untreated, and LMF-treated, respectively. A tick (V) indicates that the pathway was present for the EV proteome.

STRING Cluster Pathways	PNT2 ctrl	PNT2 LMF
Formation of the cornified envelope and Autosomal recessive congenital ichthyosis	V	
Formation of the cornified envelope and Serpin, conserved site	V	V
Desmosome and Ichthyosis vulgaris	V	V
Keratinisation and Cornified envelope	V	
Mixed, incl. Pachyonychia congenita and Epidermolysis bullosa simplex Dowling–Meara type	V	
Pachyonychia congenita and Epidermolysis bullosa simplex Dowling–Meara type	V	V
Mixed, incl. Pachyonychia congenita and Netherton syndrome	V	
Mixed, incl. Ichthyosis vulgaris and Bullous congenital ichthyosiform erythroderma	V	
Pachyonychia congenita and Epidermolysis bullosa simplex Dowling–Meara type	V	V
Mixed, incl. S100/CaBP-9k-type, calcium binding, subdomain, and Cystatin superfamily	V	
Naxos disease and Subcorneal pustular dermatosis	V	
Ichthyosis vulgaris and Epidermolytic acanthoma	V	V
S100/CaBP-9k-type, calcium binding, subdomain, and Annexin	V	
S-100/ICaBP-type calcium binding domain	V	
Keratin filament and Keratin, type I	V	
Detoxification of ROS and mRNA, protein, and metabolite induction pathway by cyclosporin A	V	
Mixed, incl. COVID-19, thrombosis and anticoagulation, and Scavenging of heme from plasma	V	
Mixed, incl. Glutathione metabolism and Detoxification of Reactive Oxygen Species	V	
Alcoholic pancreatitis and Typhus	V	V
Cell adhesive protein binding involved in bundle of His cell-Purkinje myocyte communication	V	
**KEGG Pathways:**	**PNT2 ctrl**	**PNT LMF**
Oestrogen signalling pathway	V	V
*Staphylococcus aureus* infection	V	V
Biosynthesis of amino acids	V	
**Biological Process GO**	**PNT2 ctrl**	**PNT LMF**
Intermediate filament organisation	V	V
Intermediate filament cytoskeleton organisation		V
Epidermis development	V	V
Keratinocyte differentiation	V	V
Keratinisation	V	V
Skin development	V	V
Epithelial cell differentiation	V	V
Supramolecular fibre organisation	V	V
Epithelium development	V	
Tissue development	V	
Cellular oxidant detoxification	V	
Response to toxic substance	V	
Multicellular organismal process	V	
Anatomical structure development	V	V
Cell differentiation	V	
Developmental process	V	
Cytoskeleton organisation	V	V
Animal organ development	V	
Peptide cross-linking	V	
Retina homeostasis	V	
Humoral immune response	V	V
Immune response		V
Cell–cell adhesion	V	
Multicellular organismal homeostasis	V	
Antimicrobial humoral response	V	V
Response to biotic stimulus	V	
Cellular process	V	
Response to reactive oxygen species	V	
Peptidyl-cysteine S-nitrosylation	V	
Hydrogen peroxide catabolic process	V	
Catabolic process	V	
Biological process involved in interspecies interaction between organisms	V	
Response to other organism	V	
Establishment of skin barrier	V	
Cell adhesion	V	
Defence response to other organism	V	V
Immune system process	V	
Cellular catabolic process	V	
Response to external stimulus	V	V
Tissue homeostasis	V	
Homeostatic process	V	
Ageing	V	
Response to oxidative stress	V	
Glucose metabolic process	V	
Peptidyl-cysteine S-trans-nitrosylation	V	
Neutrophil aggregation	V	
Defence response to fungus	V	
Cell envelope organisation	V	
Defence response	V	
Defence response to bacterium	V	
Response to bacterium	V	
Sequestering of zinc ion	V	
**Wiki Pathways**	**PNT2 ctrl**	**PNT LMF**
Aerobic glycolysis	V	
Glycolysis in senescence	V	
Network map of SARS-CoV-2 signalling pathway	V	
Corticotropin-releasing hormone signalling pathway		V
**Molecular Function GO**	**PNT2 ctrl**	**PNT LMF**
Structural constituent of skin epidermis	V	V
Structural molecule activity	V	V
Antioxidant activity	V	
Fatty acid binding	V	
Structural constituent of cytoskeleton	V	V
Calcium ion binding	V	
**Cellular Component GO**	**PNT2 ctrl**	**PNT LMF**
Extracellular space	V	V
Extracellular exosome	V	V
Extracellular region	V	
Vesicle	V	V
Cornified envelope	V	V
Intermediate filament cytoskeleton	V	
Intermediate filament	V	V
Keratin filament	V	V
Secretory granule	V	V
ficolin-1-rich granule	V	
Secretory granule lumen	V	
Polymeric cytoskeletal fiber	V	
Cytoplasmic vesicle	V	
Desmosome	V	
Supramolecular fiber	V	
ficolin-1-rich granule lumen	V	
Cytosol	V	V
Tertiary granule	V	
Cytoskeleton	V	V
Cytoplasm	V	
Melanosome	V	
Collagen-containing extracellular matrix	V	V
Blood microparticle	V	V
Tertiary granule lumen	V	
ficolin-1-rich granule membrane	V	
Vacuolar lumen	V	
Endomembrane system	V	
Azurophil granule lumen	V	
Organelle	V	
Lysosome	V	
Specific granule lumen	V	
Endocytic vesicle lumen	V	V
Keratohyalin granule	V	
Membrane-bounded organelle	V	
Intracellular organelle	V	
Intracellular non-membrane-bounded organelle	V	V
Intracellular anatomical structure	V	
Cell periphery	V	
Cell–cell junction	V	
Proteasome core complex, alpha-subunit complex	V	
Fascia adherens	V	
Secretory granule membrane	V	
Haptoglobin–haemoglobin complex	V	
Cytoplasmic vesicle membrane	V	
Haemoglobin complex	V	
Bounding membrane of organelle	V	
Endocytic vesicle	V	
**Disease–Gene Associations**	**PNT2 ctrl**	**PNT LMF**
Keratosis	V	V
Palmoplantar keratosis	V	V
Integumentary system disease	V	
Skin disease	V	V
Amyloidosis	V	
Pachyonychia congenita	V	V
Familial visceral amyloidosis	V	
Nonepidermolytic palmoplantar keratoderma	V	V
Autosomal dominant disease	V	V
Bullous skin disease	V	V
Dermatitis	V	
Acanthoma	V	V
Pemphigus	V	
Steatocystoma multiplex	V	V
Borst–Jadassohn intraepidermal carcinoma	V	V
Subcorneal pustular dermatosis	V	
Carcinoma	V	V
Epidermolysis bullosa	V	
Seborrheic keratosis	V	V
Mycosis fungoides	V	V
Basal cell carcinoma	V	V
Skin cancer	V	
Hair disease	V	
Ichthyosis	V	
Inherited metabolic disorder	V	
Autosomal genetic disease	V	V
Disease	V	
Genetic disease	V	
Monogenic disease	V	
Arrhythmogenic right ventricular cardiomyopathy	V	
Primary cutaneous amyloidosis	V	V
Epidermolysis bullosa simplex Dowling–Meara type	V	V
Epidermolysis bullosa simplex with mottled pigmentation	V	V
Focal nonepidermolytic palmoplantar keratoderma	V	V
Epidermolytic acanthoma	V	V
Bullous congenital ichthyosiform erythroderma	V	V
Disease of anatomical entity	V	V
Immune system disease	V	
Epidermolytic hyperkeratosis	V	V
Stomach cancer	V	
Naxos disease	V	
Autoimmune disease of skin and connective tissue	V	
Hematopoietic system disease		V
**Reactome Pathways**	**PNT2 ctrl**	**PNT LMF**
Formation of the cornified envelope	V	V
Neutrophil degranulation	V	
Developmental Biology	V	
Innate Immune System	V	
Immune System	V	
Cellular response to chemical stress	V	
Scavenging of heme from plasma	V	V
Cellular responses to stress	V	
Chaperone-Mediated Autophagy	V	
Apoptotic cleavage of cell adhesion proteins	V	
Antimicrobial peptides	V	
ER-Phagosome pathway	V	
Apoptosis	V	
Transport of small molecules	V	
The role of GTSE1 in G2/M progression after G2 checkpoint	V	
Metal sequestration by antimicrobial proteins	V	
Detoxification of Reactive Oxygen Species	V	
PCP/CE pathway	V	
Class I MHC-mediated antigen processing and presentation	V	
RUNX1 regulates transcription of genes involved in differentiation of HSCs	V	
Erythrocytes take up oxygen and release carbon dioxide	V	
Transport of fatty acids	V	
Type I hemidesmosome assembly		V
Cell junction organisation		V
Uptake of dietary cobalamins into enterocytes		V
**Human Phenotype (Monarch)**	**PNT2 ctrl**	**PNT LMF**
Abnormal blistering of the skin	V	V
Palmoplantar keratoderma	V	V
Hyperkeratosis	V	
Nail dystrophy	V	V
Palmoplantar blistering	V	V
Abnormal epidermal morphology	V	
Epidermal thickening	V	
Blistering by anatomical location	V	V
Epidermal acanthosis	V	V
Follicular hyperkeratosis	V	V
Abnormality of the nail	V	
Hypohidrosis or hyperhidrosis	V	V
Alopecia	V	V
Nail dysplasia	V	V
Cheilitis	V	
Angular cheilitis	V	V
Hyperhidrosis	V	V
Onychogryphosis	V	V
Abnormal hair quantity	V	V
Erythema	V	V
Lower limb pain	V	V
Scaling skin	V	V
Abnormality of skin physiology	V	
Natal tooth	V	V
Steatocystoma multiplex	V	V
Eruptive vellus hair cyst	V	V
Linear arrays of macular hyperkeratosis in flexural areas	V	V
Neoplasm of the skin	V	V
Abnormality of the skin	V	
Onychogryphosis of toenails	V	V
Abnormality of skin morphology	V	V
Hyperplastic callus formation	V	V
Recurrent skin infections	V	V
Pruritus	V	
Onychogryphosis of fingernail	V	V
Abnormal fingernail morphology	V	V
Inflammatory abnormality of the skin	V	V
Abnormal oral mucosa morphology	V	V
Paronychia	V	V
Fragile skin	V	V
Palmoplantar hyperhidrosis	V	V
Epidermoid cyst	V	V
Hoarse voice	V	V
Absent toenail	V	V
Ear pain	V	V
Fingernail dysplasia	V	V
Abnormality of the respiratory system	V	V
Abnormality of immune system physiology	V	V
Abnormality of the dentition	V	V
Sparse hair	V	
Erythroderma	V	
Oral leukoplakia	V	V
White lesion of the oral mucosa	V	V
Skin erosion	V	V
Increased inflammatory response	V	
Decreased body weight	V	V
Localised skin lesion	V	V
Acantholysis	V	
Parakeratosis	V	
Constitutional symptom	V	V
Aplasia cutis congenita	V	V
Abnormality of the immune system	V	
Generalised abnormality of skin	V	V
Cutaneous photosensitivity	V	V
Pain in head and neck region	V	V
Neoplasm by anatomical site	V	V
Alopecia universalis	V	
Orthokeratosis	V	
Abnormality of the hand	V	
Abnormality of the lower limb	V	
Sepsis	V	
Onycholysis	V	
Failure to thrive	V	
Abnormality of the face	V	V
Sign or symptom	V	
Pain	V	V
Abnormality of nail colour	V	
Recurrent infections	V	
Abnormality of metabolism/homeostasis	V	
Growth abnormality	V	V
Mottled pigmentation of the trunk and proximal extremities	V	V
Discrete 2 to 5 mm hyper- and hypopigmented macules	V	V
Palmoplantar scaling skin	V	
Abnormal circulating transferrin concentration	V	
Unusual infection	V	
Abnormality of limbs	V	
Abnormal oral cavity morphology	V	
Impaired myocardial contractility	V	
Congenital bullous ichthyosiform erythroderma	V	V
Focal friction-related palmoplantar hyperkeratosis	V	V
Localised epidermolytic hyperkeratosis	V	
Generalised reticulate brown pigmentation	V	V
Abnormal immunoglobulin level	V	
Congenital ichthyosiform erythroderma	V	
Pain	V	V
Congenital alopecia totalis	V	
Punctate palmoplantar hyperkeratosis	V	V
Skin fragility with non-scarring blistering	V	V
Widely spaced toes	V	
Smooth tongue	V	V
4–5 finger syndactyly	V	
Abdominal symptom	V	V
Foot pain	V	V
Genital blistering	V	V
Chapped lip	V	
Hyperkeratotic papule	V	V
Phenotypic abnormality	V	V
Skin plaque	V	V
Acute episodes of neuropathic symptoms	V	V
Reticulated skin pigmentation	V	V
Depigmentation/hyperpigmentation of skin	V	
Aplasia cutis congenita on trunk or limbs	V	V
Hypomelanotic macule	V	V
Tapered distal phalanges of finger	V	
Hypovolemic shock	V	
Abnormal circulating metabolite concentration	V	
Abnormal circulating protein concentration	V	V
Aplasia/Hypoplasia of the eyebrow	V	
Abnormality of hair texture	V	
3–4 finger syndactyly	V	
Erythematous papule	V	V
Obsolete Bilateral external ear deformity	V	
Abnormal cellular phenotype	V	
Hypernatremia	V	
Patchy palmoplantar hyperkeratosis	V	
Upper limb pain	V	V
Abnormality of digestive system physiology	V	V
Sparse scalp hair	V	
Spotty hyperpigmentation	V	V
Spotty hypopigmentation	V	
Diffuse palmoplantar hyperkeratosis	V	V
Right ventricular cardiomyopathy	V	
Erosion of oral mucosa	V	V
Increased neuronal autofluorescent lipopigment	V	
Lamina lucida cleavage	V	V
Mitten deformity	V	
Autoamputation of digits	V	
Abnormal dermoepidermal hemidesmosome morphology	V	
Abnormality of the digestive system		V
Abnormality of the skeletal system		V
Abnormality of the musculoskeletal system		V
Abnormal musculoskeletal physiology		V
Conjunctival hamartoma		V
Abnormal skeletal morphology		V
Oral mucosal blisters		V
Heat intolerance		V
Squamous cell carcinoma of the skin		V
Abnormal conjunctiva morphology		V
Bronchomalacia		V
Milia		V
Abnormal epiglottis morphology		V
Abnormality of temperature regulation		V
Ridged nail		V
Dystrophic toenail		V
Distal lower limb amyotrophy		V
Atrophic scars		V
Respiratory distress		V
Poor appetite		V

## Data Availability

The original contributions presented in the study are included in the article/Supplementary Materials, further inquiries can be directed to the corresponding authors.

## Supplementary Materials

The following supporting information can be downloaded at: https://www.mdpi.com/article/10.3390/biology13090734/s1, Figure S1. Full western blot images for CD63 and Flot-1 on EVs. Table S1. PC3 control EV proteomes by LC-MS/MS analysis. Table S2. PC3 4 h LMF-treated EV proteomes by LC-MS/MS analysis. Table S3. PNT2 control EV proteomes by LC-MS/MS analysis. Table S4. PNT2 4 h LMF-treated EV proteomes by LC-MS/MS analysis.
