# Supplementary figures and images for: Low Magnetic Field Exposure Alters Prostate Cancer Cell Properties
Source: Biology (Basel). 2024 Sep 19;13(9):734. doi: 10.3390/biology13090734 (PMC11428832; doi:10.3390/biology13090734)

**FIGURE 1E.**

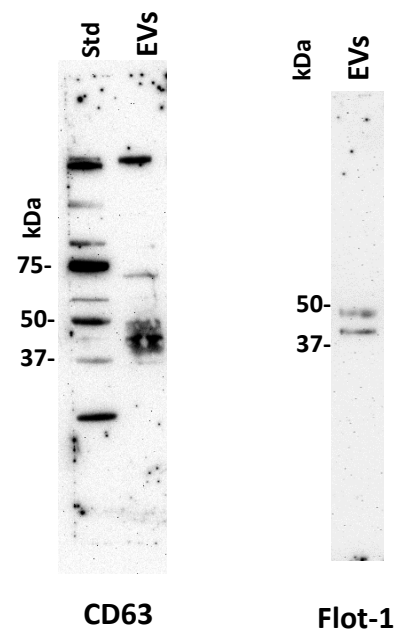

Supplement: Supplementary file 1 [file biology-13-00734-s001.zip › Figure S1.pdf]
